# Antiparasitic Effect of Polyphenols and Terpenes from Natural Products Against *Trypanosoma cruzi* and *Leishmania mexicana*

**DOI:** 10.3390/metabo15080560

**Published:** 2025-08-21

**Authors:** Diana V. Navarrete-Carriola, Gildardo Rivera, Eyra Ortiz-Pérez, Alma D. Paz-González, Ana Verónica Martínez-Vázquez, Laura Victoria Aquino-González, Liliana Argueta-Figueroa, Michael P. Doyle, Adriana Moreno-Rodríguez

**Affiliations:** 1 Laboratorio de Biotecnología Farmacéutica, Centro de Biotecnología Genόmica, Instituto Politécnico Nacional, Reynosa 88710, Mexico; civky82@gmail.com (D.V.N.-C.); giriveras@ipn.mx (G.R.); eortizp@ipn.mx (E.O.-P.); apazg@ipn.mx (A.D.P.-G.); avmartinez@ipn.mx (A.V.M.-V.); 2 Department of Chemistry, The University of Texas at San Antonio, San Antonio, TX 78249, USA; 3 Laboratorio de Estudios Epidemiológicos, Clínicos, Diseños Experimentales e Investigación, Facultad de Ciencias Químicas, Universidad Autónoma “Benito Juárez” de Oaxaca, Oaxaca 68120, Mexico; augl770623.fmc@uabjo.mx; 4 SECIHTI—Tecnológico Nacional de México, Instituto Tecnológico de Toluca, Metepec 52149, Mexico; liliana.argueta@secihti.mx

**Keywords:** natural products, polyphenols, terpenes, secondary metabolites, *T. cruzi*, *L. mexicana*

## Abstract

**Background**: Worldwide, the number of cases of parasitic diseases has been increasing; however, available treatments have variable adverse effects and low efficacy, mainly in Neglected Tropical Diseases such as Chagas disease and Leishmaniasis. Therefore, the development of new and more effective antiparasitic drugs is important. Natural products are the source of secondary metabolites with different biological activities, such as antibacterial, anticancer, anti-inflammatory, and antiparasitic. **Objectives**: In this work, secondary metabolites (phenols and terpenes) from natural products were selected to be evaluated against the epimastigotes of NINOA and A1 strains of *Trypanosoma cruzi* and the promastigotes of M379 strain and FCQEPS native isolate of *Leishmania mexicana*. Additionally, their cytotoxicity and selectivity index were determined. **Methods**: Eighteen secondary metabolites were evaluated in vitro against *T. cruzi* epimastigotes and *L. mexicana* promastigotes; additionally, their cytotoxicity on the J774.2 macrophage cell line was determined. **Results**: The compounds l-(-)-menthol (14, IC_50_ = 24.52 µM) and β-citronellol (11, IC_50_ = 21.54 µM) had higher trypanocidal activity than the reference drug (benznidazole) against NINOA and A1 strains of *T. cruzi*, respectively. On the other hand, *para*-anisyl alcohol (4, IC_50_ = 34.89 µM) had higher leishmanicidal activity than the reference drug (glucantime^®^) against M379 and the FCQEPS native isolate of *L. mexicana*. Finally, in silico, the determination of their pharmacokinetic and toxicological properties showed that they are promising candidates for oral and topical uses. **Conclusions**: This study opens the possibility of using secondary metabolites as scaffolds for access to the development of new molecules for the treatment of parasite diseases.

## 1. Introduction

Currently, the number of cases of parasitic diseases, estimated in 2015 as 48.4 million cases and 59,724 deaths annually, is increasing by 8.78 million new cases per year [1]. Some of them are considered Neglected Tropical Diseases, highlighting those caused by the trypanosomatidae parasites, *Trypanosoma cruzi*, and *Leishmania* spp.

Chagas disease (CD) is caused by *T. cruzi*, which affects between 6 and 7 million people worldwide, with approximately 12,000 deaths each year. Transmission is mainly related to the presence of the vector (triatomine bugs, commonly referred to as “kissing bugs”), although blood transfusion and ingestion of contaminated food are other pathways of infection. Today, approximately 75 million people are at risk of infection in the 44 countries where CD is found, but only 6 of these countries have systems in place to monitor cases and transmission. CD is responsive to only two drugs, benznidazole or nifurtimox, which are effective in curing the disease if they are administered at the beginning of the acute phase, even in cases of congenital transmission [2,3,4]. However, these drugs cause several adverse effects and have variable efficacy in the chronic stage.

Leishmaniasis is a wide array of clinical manifestations caused by protozoal parasites of the genus *Leishmania*; an estimated 700,000 to 1 million new cases occur annually [5]. There are three forms of leishmaniasis: visceral (the most serious form because it is almost always fatal without treatment), cutaneous (the most common, usually causing skin ulcers), and mucocutaneous (affecting the mouth, nose, and throat) [5,6]. Leishmania parasites are transmitted through the bites of infected female phlebotomine sandflies, which feed on blood to produce eggs. The World Health Organization (WHO) recommends pentavalent antimonials, which have side effects and low effectiveness, as first-line drug treatment [5,6]. However, despite the availability of these pharmacological treatments, new drugs need to be developed [7].

On the other hand, natural products (NPs) are compounds that can be obtained from various naturally occurring sources. Traditionally, medicinal plants are the most important source of a wide variety of bioactive compounds known as secondary metabolites [8,9,10]. These compounds have an essential role in the search for new drugs, providing scaffolds for drug discovery and development.

**Figure 1:**
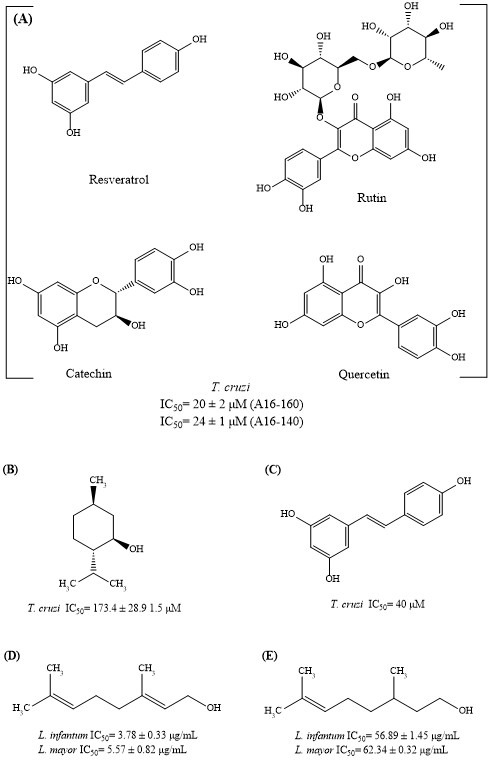
Secondary metabolites (terpenoids and phenolic compounds) from medicinal plants with antiprotozoal activity. (**A**): resveratrol, rutin, catechin, and quercetin, (**B**): dl-menthol, (**C**): resveratrol, (**D**): geraniol and (**E**): citronellol. The figure is created with ChemDraw 22.0.0.

The secondary metabolites of plants fall into three main categories: (a) terpenols and terpenoids, (b) alkaloids, and (c) phenolic compounds. These secondary metabolites have different biological effects, including anticancer, antibacterial, antioxidant, anti-inflammatory, anti-hyperglycemic, and antiparasitic activities [11,12]. A wide range of polyphenols have activity against parasites that include *Acanthamoeba*, *Caenorhabditis elegans*, *Toxoplasma gondii*, *Haemonchus* spp., *Teladorsagia circumcincta*, *Trichostrongylus colubriformis*, *Trypanosoma cruzi*, and *Leishmania* sp. [13,14,15,16]. For example, extracts encapsulated with different polyphenols (resveratrol, rutin, catechin, and quercetin, Figure 1a) have activity against *T. cruzi* (IC_50_ < 100 µg/mL) [17]. Dl-menthol (IC_50_ = 173 µM) and resveratrol (IC_50_ = 40 µM) had a trypanocidal activity against *T. cruzi* (Figure 1b,c) [18,19], and geraniol (Figure 1d), as well as citronellol (Figure 1e), had leishmanicidal activity against *L. infantum* (causing visceral leishmaniasis) and *L. major* (causing cutaneous leishmaniasis) [20]. These results contribute to the identification of new molecules with potential antiparasitic activity and allow the design of new drugs to combat different diseases. Therefore, in this study, secondary metabolites of the terpene and polyphenol families were evaluated in vitro against two strains of *T. cruzi* and *L. mexicana* as potential antiparasitic agents. Additionally, their cytotoxicity on the J774.2 macrophage cell line was determined.

## 2. Materials and Methods

### 2.1. Reagents

Compounds of the screening set were commercially acquired (1–3, 6, 7, 9–11, 15, and 16) from Sigma-Aldrich (Toluca, Mexico)^®^; 5, 8, 12, 13, 17, and **18** from TCI AMERICA (Portland, OR, USA); 4 from Oakwood Chemical (Estill, SC, USA); and 14 from Across Organics (Medley, FL, USA) and were used without further purification.

### 2.2. Biological Assays

#### 2.2.1. Anti-T*rypanosoma cruzi* Activity

Epimastigotes from A1 and the NINOA strains were used to evaluate the trypanocidal activity. Both strains were maintained in liver infusion tryptose (LIT) medium, supplemented with 10% heat-inactivated FBS and 100 U/mL of penicillin plus 100 μg/mL of streptomycin. They were preserved by transferring 1 × 10^6^ parasites/mL into a new culture medium every week. The activity of natural products, as well as nifurtimox and benznidazole, was evaluated against *T. cruzi* strains. All compounds were initially prepared at 10 mg/mL, using dimethyl sulfoxide (DMSO) 0.2% as diluent. Serial dilutions were performed using complemented LIT medium until concentrations of 200–0.78 µM of each drug were obtained. *T. cruzi* epimastigotes (1 × 10^6^/well) were cultured in 96-well microliter plates and incubated for 48 h at 28 °C with the drugs at different concentrations in a final volume of 200 μL. DMSO 0.2% was included as a negative control. The metabolic activity of the cells was determined using the method of 3-(4,5-dimethylthiazol-2-yl)-2,5-diphenyltetrazolium bromide (MTT). All assays were carried out in triplicate. The IC_50_ values were determined by Probit analysis. The data were the percentage of viability of the parasite to the different concentrations of secondary metabolites used; these data are transformed into a linear form on which the regression analysis is performed [21,22,23,24].

#### 2.2.2. Anti-*Leishmania mexicana* Activity

Promastigotes from the MNYC/BZ/62/M379 (M379) reference strain and the (MHOM/MX/2018/UABJOFCQEPS (FCQEPS) native isolate of *L. mexicana* were used for leishmanicidal activity [21]. Parasites were preserved in RPMI 1640 culture medium supplemented with 10% heat-inactivated FBS and 100 U/mL of penicillin plus 100 μg/mL of streptomycin, and glutamine (2 mM). Promastigotes (5 × 10^5^ parasites/mL) were added to a 96-well plate, then 200 μL of each compound at different concentrations (200–0.78 μM) dissolved in DMSO in a final volume of 200 μL for 48 h at 26 °C. Promastigotes treated with DMSO 0.2% were used as negative controls, and the reference drug glucantime was included as a positive control. The metabolic activity of the cells was determined using the method of 3-(4,5-dimethylthiazol-2-yl)-2,5-diphenyltetrazolium bromide (MTT). The IC_50_ was determined using the Probit statistical tool. Each sample was assayed in triplicate in three independent experiments [21,22,23,24].

#### 2.2.3. Cytotoxic Analysis Against J774.2 Macrophages

The assay was performed on mouse macrophages from the J774.2 cell line that were cultured in RPMI medium supplemented with 10% SFB, 100 U µg/mL penicillin, 100 mg/mL streptomycin, and glutamine (2 mM) at 37 °C and in a 5% CO_2_ atmosphere. The medium was replaced at 2–3-day intervals. The cells were incubated with different concentrations of the compounds 1–18 (200–0.78 µM), incubated for 48 h at 37 °C and 5% CO_2_ atmosphere. Cells in the presence of the maximum concentration of DMSO (0.2%) were included as a negative control; the metabolic activity of the cells was determined following the MTT method. The percentage of cell viability was calculated, and the half-maximal cytotoxic concentration (CC_50_) was determined by Probit analysis. Three independent assays were performed in triplicate each [21].

### 2.3. ADMET In Silico

The in silico analysis of the ADMET and cytotoxicity properties of the secondary metabolites used in this study was performed on the SwissADME platform (http://www.swissadme.ch/ accessed on 15 February 2025) for physicochemical and pharmacokinetic properties and on the ProTox 3.0 platform (ProTox-3.0—Prediction of TOXicity of chemicals) for cytotoxicity predictions.

## 3. Results

### 3.1. Small Library of Natural Products

**Figure 2:**
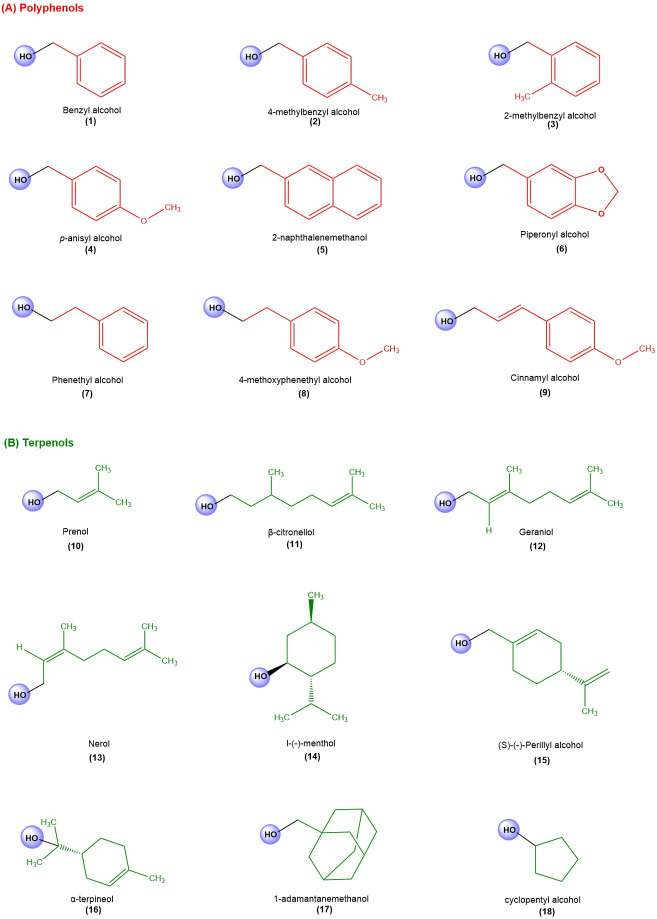
Structure of secondary metabolites belonging to the family of polyphenols (**A**) and terpenoids (**B**). The figure is created with ChemDraw 22.0.0.

According to previous reports on the antiprotozoal activity of natural products, a total of eighteen different secondary metabolites (Figure 2) belonging to the polyphenol and terpene families were selected in this study.

### 3.2. Antiparasitic Activity

The secondary metabolites were selected to be evaluated in vitro against *T. cruzi* epimastigotes and *L. mexicana* promastigotes to determine the IC_50_ values, as well as to determine the CC_50_ in macrophages. (Table 1 and Table 2). Additionally, the reference drugs benznidazole, nifurtimox, and glucantime were used as positive controls. All the experiments were evaluated in triplicate.

### 3.3. ADMET In Silico Properties

Finally, an in silico analysis was conducted to evaluate the physicochemical, pharmacokinetic (Swiss ADME), and cytotoxicity (ProTox 3.0) molecular properties of the secondary metabolites. The results are shown in Table 3, Table 4 and Table 5. The physicochemical parameters correspond to molecular weight, rotatable bonds, hydrogen bonds (acceptors and donors), polar surface area (TPSA), partition coefficient (Log P), and solubility coefficient (Log S). In terms of pharmacokinetic parameters, gastrointestinal absorption, permeability of the blood–brain barrier, P-glycoprotein (P-gp) substrate, and various isoforms of cytochrome P450 (1A2, 2C19, 2C9, 2D6), and cytotoxicity predictions such as hepatotoxicity, carcinogenicity, mutagenicity, and cytotoxicity.

## 4. Discussion

### 4.1. Antiparasitic Activity

#### 4.1.1. Trypanocidal Activity

The polyphenol family (1**–**9) had different behavioral activities against *T. cruzi* strains (Table 1). Against the NINOA strain, benzyl alcohol (1) had null activity (IC_50_ >200 µM), but the incorporation of a methyl group at the *para* position of benzyl alcohol (2) improved activity (IC_50_ = 51.85 µM), however, a change of the methyl group from the *para* to the *ortho* position (3) caused a loss of activity (IC_50_ > 200 µM) and the incorporation of a methoxy group at the *para* (4) position did not improve the trypanocidal activity (IC_50_ = 118.75 µM). 2-naphtalenemethanol (5) had better activity (IC_50_ = 118.75 µM) than piperonyl alcohol (**6**) (IC_50_ > 200 µM). Finally, phenethyl alcohol (7) increases the activity (IC_50_ = 48.24 µM). However, 4-methoxyphenethyl alcohol (8) and cinnamyl alcohol (9) had no biological activity.

In the terpenol family (10**–**18), prenol (10) had higher activity (IC_50_ = 27.33 µM) than the reference drug benznidazole (IC_50_ = 39.08 µM). However, increasing the number of carbons (β-citronellol: 11) or the number of double bonds (geraniol: 12) causes a decrease in trypanocidal activity. The *trans* isomer had higher activity (12, IC_50_ = 41.04 µM) than did the *cis* isomer (nerol: 13, IC_50_ = 58.30 µM). Finally, the cyclic terpenols 15 to 18 had no activity (IC_50_ > 200 µM), except for l-(-)-menthol (14) (IC_50_ = 24.52 µM), which had higher trypanocidal activity than the reference drug benznidazole.

Against the A1 strain, the phenolic compounds had variable trypanocidal activities similar to those of the NINOA strain; benzyl alcohol (1) and 4-methylbenzyl alcohol (2) had medium activity (IC_50_ = 62.28 and 50.43 µM, respectively). 2-methylbenzyl alcohol (3) was inactive (IC_50_ > 200 µM) against both strains. The addition of a methoxy group (p-anisyl alcohol: 4) caused a decrease in the activity (IC_50_ = 118.75 µM). Compounds 3**, **5**–**8 were inactive (IC_50_ >200 µM). On the other hand, cinnamyl alcohol (9), with a C=C bond, exhibited an increase in the trypanocidal activity (IC_50_ = 22.12 µM), surpassing the reference drug benznidazole (IC_50_ = 30.3 µM).

For compounds of the terpene family, compound **10** had no biological effects (IC_50_ > 200 µM), but increasing the number of carbons increased (β-citronellol, 11), activity improved notably (IC_50_ = 21.54 µM), surpassing the reference drug benznidazole. However, an increase in the number of double bonds in the terpene chain (geraniol, 12) caused a decrease in the activity (IC_50_ = 124.51 µM), and the *cis* isomer had higher activity (IC_50_ = 65.66 µM) than did the *trans* isomer (**13**, IC_50_ = 124.51 µM).

The cyclic terpenols 14, 15, and 17 had no biological activity. Previously, l-(-)-menthol (14) was reported with null trypanocidal activity against the Tulahuén strain of *T. cruzi* [18]; only α-terpineol (16, IC_50_ = 14.02 µM) and cyclopentyl alcohol (18, IC_50_ = 10.83 µM) had better activity than the reference drug benznidazole (IC_50_ = 30.3 µM), but lower than nifurtimox (IC_50_ = 7.09 µM). Unfortunately, cyclopentyl alcohol has a high toxicity (CC_50_ = 24.42 µM).

Interestingly, compounds 2, 11, and 13 had broad-spectrum activity against both strains; therefore, these compounds could be considered for developing new and more potent anti-*T. cruzi* agents.

#### 4.1.2. Leishmanicidal Activity

The phenols and terpenols derivatives also had different activities against both strains of *L. mexicana* (Table 2). Against the M379 strain, benzyl alcohol (1) and 4-methylbenzyl alcohol (2) had no biological effects (IC_50_ > 200 µM); however, with the methyl group at the *ortho*-position (2-methylbenzyl alcohol, 3), a notable increase in the leishmanicidal activity (IC_50_ = 14.23 µM) was observed. Also, compounds 4–7 and 9 had high activity (IC_50_ < 40.10 µM); however, with a *para*-methoxy group (8), only medium activity (IC_50_ = 52.08 µM) was achieved. These results can be interpreted as an increase in the affinity for membrane lipids of the parasite for compounds having electron-donating groups (EDG), affecting cellular transport, or facilitating the entry of the molecule into the parasite [25]. Compounds 3–7 had the highest leishmanicidal activity compared to the reference drug Glucantime; however, 2-methylbenzyl alcohol (**3**) and cinnamyl alcohol (9) had high cytotoxicity.

In the terpenol family, prenol (10) did not have leishmanicidal activity, while compounds β-citronellol (11), geraniol (12), and *l-(-)-*menthol 14 had medium activity with IC_50_ values from 57.0 to 81.46 µM, with the *trans*- isomer (geraniol, 12) having higher activity than the *cis*- (nerol, **13**) isomer (IC_50_ = 106.40 µM). In chalcone secondary metabolites, the *cis*-isomers of certain derivatives were reported to have better leishmanicidal (*L. infantum*) and trypanocidal activity, because the *cis*-isomers adopt more favorable conformations to bind to essential proteins of the parasite; however, the trans isomer is usually less toxic, more stable, and improves bioavailability [26]. Finally, the cyclic terpenols 15–18 proved to be the most active (IC_50_ < 35.09 µM); however, compounds 16–18 had high cytotoxicity.

Against the FCQEPS native isolate, 1 had a low leishmanicidal activity (IC_50_ = 104.01 µM), and 2 was inactive (IC_50_ > 200 µM), although incorporation of the methyl group at the *ortho*- position on the phenyl ring (3) increased substantially the leishmanicidal activity (IC_50_ = 19.09 µM), suggesting that steric effects are key factors in biological activity. However, the addition of a more polar group (methoxy, 4) decreases activity by a factor of two.

Compound 5 with the naphthalene ring also had low activity (IC_50_ = 125.37 µM). In contrast, compounds such as phenethyl alcohol (7) or cinnamyl alcohol (9) had high activity (IC_50_ = 42.24 µM; IC_50_ = 29.06 µM, respectively); however, both compounds had high cytotoxicity.

In the terpenol family, compounds 10–15 did not exhibit biological effects (IC_50_ > 200 µM), while cyclopentyl alcohol (18) had low activity (IC_50_ = 125.37 µM). The cyclic terpenes α-terpineol (16) and 1-adamantanemethanol (17) exhibited an increase in activity (IC_50_ = 75.57 µM; IC_50_ = 30.20 µM). However, these compounds showed a high cytotoxicity (IC_50_ = 63.41 µM; IC_50_ = 12.69 µM).

Interestingly, compounds 4, 6, 7, 9, and 17 had broad-spectrum activity against both strains, with *p*-anisyl alcohol being the most active for both strains of *L. mexicana*; therefore, these secondary metabolites are potential scaffolds to develop new drugs against *L. mexicana*.

### 4.2. ADMET In Silico Properties

The predictive study showed that all secondary metabolites evaluated have a MW < 160 g/mol, suggesting that the possibility of absorption by passive diffusion, permeability, and bioavailability is favored. All metabolites have a very low TPSA, which predicts high intestinal absorption and permeability to the central nervous system. The log p ranges (1–3) show a balance between solubility and lipophilicity, favoring absorption and distribution. In addition, the solubility was predicted for compounds 1, 4, 5, 8–10, and 18, which are very soluble, and for compounds 2, 3, 6, 7, and 11–17, which are soluble. This property is crucial for oral bioavailability and pharmaceutical formulation. The toxicity predictions (hepatotoxicity, mutagenicity, carcinogenicity, and cytotoxicity) were negative, except for 1-adamantanemethanol (17), which is potentially carcinogenic. As for CYP450 inhibition, they showed that, according to the family, terpenes compounds do not inhibit any of the isoforms analyzed, and of the polyphenol family, compounds 1–5 and 7–9 inhibit the 1A2 isoform, which inhibition may change the plasma concentration of the drug, affecting its effective distribution (Table 3, Table 4 and Table 5). These results suggest that these secondary metabolites are promising candidates for oral use, which is important for the treatment of Chagas disease, as well as for their topical use in the treatment of cutaneous leishmaniasis.

## 5. Conclusions

In this work, an in vitro biological evaluation against *T. cruzi* and *L. mexicana* identified two secondary metabolites (11 and 4) to develop new agents against Chagas disease and Leishmaniasis, respectively. β-Citronellol (11) had higher activity against epimastigotes of the NINOA and A1 strains of *T. cruzi* than benznidazole, and *para*-anisyl alcohol (4) had the highest activity against promastigotes of the M379 and FCQEPS native isolate strains of *L. mexicana*.

Furthermore, this study showed that various secondary metabolites can be used for the treatment of parasitic diseases, as well as the use of these scaffolds for access to the development of new molecules. Finally, the use of secondary metabolites from natural products can provide access to new therapeutic strategies not only for parasitic diseases involving *T. cruzi* or Leishmania, but also for the treatment of other types of parasitic diseases caused by other protozoa, which are more accessible, effective, and have better safety profiles.

## Figures and Tables

**Table 1:** Trypanocidal activity (IC_50_ in μM) of secondary metabolites against *T. cruzi* epimastigotes and their cytotoxicity and selectivity index.

Secondary Metabolites	*T. cruzi*				Cytotoxicity CC_50_ J774.2
	IC_50_ (µM)		SI		
	NINOA	A1	NINOA	A1	
(1)	>200	62.28 ± 0.2	0.1	0.4	26.46 ± 2.1
(2)	51.85 ± 0.01	50.43 ± 1.8	3.9	4.0	>200
(3)	>200	>200	0.3	0.3	64.20 ± 0.2
(4)	118.75 ± 2.9	79.49 ± 0.3	1.7	2.5	>200
(5)	124.92 ± 3.2	>200	1.6	1.0	>200
(6)	>200	>200	0.3	0.3	62.18 ± 3.2
(7)	48.24 ± 0.3	>200	1.9	0.4	89.63 ± 0.0
(8)	>200	>200	1.0	1.0	>200
(9)	>200	22.12 ± 0.2	0.2	2.1	47.35 ± 0.1
(10)	27.33 ± 1.0	>200	7.3	1.0	>200
(11)	53.57 ± 3.3	21.54 ± 0.2	3.7	9.3	>200
(12)	41.04 ± 1.2	124.51 ± 5.5	4.9	1.6	>200
(13)	58.30 ± 0.3	65.66 ± 3.1	3.4	3.0	>200
(14)	24.52 ± 0.7	>200	8.2	1.0	>200
(15)	>200	>200	1.0	1.0	>200
(16)	>200	14.02 ± 0.2	0.3	4.5	63.41 ± 0.9
(17)	>200	>200	0.1	0.1	12.69 ± 0.5
(18)	>200	10.83 ± 0.1	0.1	2.3	24.42 ± 3.2
Nfx	19.30 ± 0.1	7.09 ± 0.1	8.5	23.1	164.20 ± 0.2
Bzn	39.08 ± 0.1	30.3 ± 0.03	3.4	4.4	133.90 ± 0.06

SI = Selectivity index (CC_50_/IC_50_); Nfx: Nifurtimox; Bzn: Benznidazole.

**Table 2:** Leishmanicidal activity (IC_50_ in μM) of secondary metabolites against *L. mexicana* promastigotes and their cytotoxicity and selectivity index.

Secondary Metabolites	*L. mexicana*				Cytotoxicity CC_50_ J774.2
	IC_50_ (µM)		SI		
	M379	FCQEPS	M379	FCQEPS	
(1)	>200	104.01 ± 0.3	0.1	0.2	26.46 ± 2.1
(2)	>200	>200	1.0	1.0	>200
(3)	14.23 ± 0.2	92.97 ± 0.2	4.5	0.7	64.20 ± 0.2
(4)	34.89 ± 3.0	19.09 ± 0.4	5.7	10.4	>200
(5)	40.10 ± 4.7	>200	5.0	1.0	>200
(6)	23.61 ± 0.2	49.11 ± 0.1	2.6	1.2	62.18 ± 3.2
(7)	21.70 ±1.7	42.24 ± 0.1	4.1	2.1	89.63 ± 0.0
(8)	52.08 ± 0.5	>200	3.8	1.0	>200
(9)	12.07 ± 0.2	29.06 ± 0.1	3.9	1.6	47.35 ± 0.1
(10)	>200	>200	1.0	1.0	>200
(11)	70.98 ± 1.0	>200	2.8	1.0	>200
(12)	57.00 ± 5.1	>200	3.5	1.0	>200
(13)	106.40 ± 6.0	>200	1.9	1.0	>200
(14)	81.46 ± 4.7	>200	2.5	1.0	>200
(15)	34.42 ± 2.8	>200	5.8	1.0	>200
(16)	14.04 ± 0.2	75.57 ± 0.03	4.5	0.8	63.41 ± 0.9
(17)	35.09 ± 0.2	30.20 ± 0.2	0.3	0.4	12.69 ± 0.5
(18)	5.48 ± 0.1	125.37 ± 0.2	4.4	0.1	24.42 ± 3.2
Glu	>200	133.96 ± 4.3	1.3	2.0	>273.2

SI = Selectivity index (CC_50_/IC_50_); Glu: Glucantime.

**Table 3:** Calculated physicochemical, pharmacokinetic, and toxicity parameters of secondary metabolites 1-6 by SwissADME and ProTox 3.0.

		Compounds					
		**1**	**2**	**3**	**4**	**5**	**6**
Physicochemical	MW (g/mol)	108.1	122.2	122.2	136.2	158.2	152.1
	Rotatable bonds	1	1	1	2	1	1
	Hydrogen bond acceptors	1	1	1	2	1	3
	Hydrogen bond donors	1	1	1	1	1	1
	TPSA (Å^2^)	20.2	20.2	20.2	29.5	20.2	38.7
	Log *P*	1.7	1.6	1.9	2.0	2.1	1.9
	Log *S*	Very soluble	Soluble	Soluble	Very soluble	Soluble	Very soluble
Pharmacokinetic	GI Absorption	High	High	High	High	High	High
	Permeability BBB	Yes	Yes	Yes	Yes	Yes	Yes
	P-gp subtrate	No	No	No	No	No	No
	CYP1A2 inhibitor	Yes	Yes	Yes	Yes	Yes	No
	CYP2C19 inhibitor	No	No	No	No	No	No
	CYP2C9 inhibitor	No	No	No	No	No	No
	CYP2D6inhibitor	No	No	No	No	No	No
Toxicity	Hepatoxicity	Inactive	Inactive	Inactive	Inactive	Inactive	Inactive
	Carcinogenicity	Inactive	Inactive	Inactive	Inactive	Inactive	Inactive
	Mutagenicity	Inactive	Inactive	Inactive	Inactive	Inactive	Inactive
	cytotoxicity	Inactive	Inactive	Inactive	Inactive	Inactive	Inactive

MW: molecular weight, TPSA: polar surface area, Log P: partition coefficient, Log S: solubility coefficient, Permeability BBB: permeability of the blood–brain barrier.

**Table 4:** Calculated physicochemical, pharmacokinetic, and toxicity parameters of secondary metabolites 7-12 by SwissADME and ProTox 3.0.

		Compounds					
		**7**	**8**	**9**	**10**	**11**	**12**
Physicochemical	MW (g/mol)	126.16	152.19	134.18	86.13	156.26	154.25
	Rotatable bonds	2	3	2	1	5	4
	Hydrogen bond acceptors	1	2	1	1	1	1
	Hydrogen bond donors	1	1	1	1	1	1
	TPSA (Å^2^)	20.23	29.46	20.23	20.23	20.23	20.23
	Log *P*	1.70	2.10	1.98	1.60	2.72	2.52
	Log *S*	Very soluble	Soluble	Soluble	Very soluble	Soluble	Soluble
Pharmacokinetic	GI Absorption	High	High	High	High	High	High
	Permeability BBB	Yes	Yes	Yes	Yes	Yes	Yes
	P-gp subtrate	No	No	No	No	No	No
	CYP1A2 inhibitor	Yes	Yes	Yes	No	No	No
	CYP2C19 inhibitor	No	No	No	No	No	No
	CYP2C9 inhibitor	No	No	No	No	No	No
	CYP2D6inhibitor	No	No	No	No	No	No
Toxicity	Hepatoxicity	Inactive	Inactive	Inactive	Inactive	Inactive	Inactive
	Carcinogenicity	Inactive	Inactive	Inactive	Inactive	Inactive	Inactive
	Mutagenicity	Inactive	Inactive	Inactive	Inactive	Inactive	Inactive
	cytotoxicity	Inactive	Inactive	Inactive	Inactive	Inactive	Inactive

MW: molecular weight, TPSA: polar surface area, Log P: partition coefficient, Log S: solubility coefficient, Permeability BBB: permeability of the blood–brain barrier.

**Table 5:** Calculated physicochemical, pharmacokinetic, and toxicity parameters of secondary metabolites 13-18 by SwissADME and ProTox 3.0.

		Compounds					
		**13**	**14**	**15**	**16**	**17**	**18**
Physicochemical	MW (g/mol)	154.25	156.27	152.23	154.25	166.26	86.13
	Rotatable bonds	4	1	2	1	1	0
	Hydrogen bond acceptors	1	1	1	1	1	1
	Hydrogen bond donors	1	1	1	1	1	1
	TPSA (Å^2^)	20.23	20.23	20.23	20.23	20.23	20.23
	Log *P*	2.75	2.55	2.50	2.51	2.33	1.59
	Log *S*	Soluble	Soluble	Soluble	Soluble	Soluble	Very soluble
Pharmacokinetic	GI Absorption	High	High	High	High	High	High
	Permeability BBB	Yes	Yes	Yes	Yes	Yes	Yes
	P-gp subtrate	No	No	No	No	No	No
	CYP1A2 inhibitor	No	No	No	No	No	No
	CYP2C19 inhibitor	No	No	No	No	No	No
	CYP2C9 inhibitor	No	No	No	No	No	No
	CYP2D6inhibitor	No	No	No	No	No	No
Toxicity	Hepatoxicity	Inactive	Inactive	Inactive	Inactive	Inactive	Inactive
	Carcinogenicity	Inactive	Inactive	Inactive	Inactive	Active	Inactive
	Mutagenicity	Inactive	Inactive	Inactive	Inactive	Inactive	Inactive
	cytotoxicity	Inactive	Inactive	Inactive	Inactive	Inactive	Inactive

MW: molecular weight, TPSA: polar surface area, Log P: partition coefficient, Log S: solubility coefficient, Permeability BBB: permeability of the blood–brain barrier.

## Data Availability

All data generated or analyzed during this study are included in this published article.
